# Effects of Grape Seed Extract, Vitamin C, and Vitamin E on Ethanol- and Aspirin-Induced Ulcers

**DOI:** 10.1155/2011/740687

**Published:** 2011-11-20

**Authors:** Vivian Molina Cuevas, Yazmín Ravelo Calzado, Yohani Pérez Guerra, Ambar Oyarzábal Yera, Sonia Jiménez Despaigne, Rosa Mas Ferreiro, Daisy Carbajal Quintana

**Affiliations:** Pharmacology Department, Centre of Natural Products, National Centre for Scientific Research, Avenue 25 and 158 Street Cubanacán, Havana City 6880, Cuba

## Abstract

Effects of GSE and vitamins C and E on aspirin- and ethanol-induced gastric ulcer and associated increases of lipid peroxidation in rats were compared. Two experiments were conducted. Rats were randomized into eight groups: a negative control and seven groups that received aspirin or ethanol for ulcer induction: one positive control (vehicle) and six with VC, VE, or GSE (25 and 250 mg/kg). Ulcer indexes and gastric levels of malondialdehyde (MDA) were quantified. VC, VE, and GSE (25 and 250 mg/kg) decreased aspirin, and ethanol-induced ulcers and MDA values compared with positive control group. The magnitude of aspirin ulcer reduction was comparable for all treatments, and MDA decrease with GSE was higher than with VC and tended to be greater, albeit none significantly, than with VE. GSE was more effective than VC and VE for lowering the ethanol ulcers, while the decrease of MDA levels with GSE was greater than with VC, but comparable to that achieved with VE. GSE protected against ethanol-induced gastric ulcers more effectively than VC or VE, while its protection against aspirin ulcers was comparable for all treatments. GSE produced the greatest reductions of gastric MDA in both models.

## 1. Introduction

Peptic ulcer, a common gastrointestinal pathological condition, is due to the loss of the balance between aggressive and defensive factors of the gastric and duodenal mucosa. Aggressive factors against gastric mucosa include acid, pepsin, *Helicobacter pylori*, nonsteroidal anti-inflammatory drugs (NSAIDs), and ethanol, while local mucosal defensive factors include bicarbonate, mucus secretion, blood flow, cellular regeneration, and endogenous protective agents like prostaglandins (PG) and epidermal growth factors [1–3]. Also, increased oxidative stress is believed to be linked to the aggressive factors-induced gastric mucosal damage [4, 5]. 

The pathogenesis of NSAIDs-induced gastric ulceration includes the block of cyclooxygenase (COX) activity that leads to lower mucus and bicarbonate secretion, decreased mucosal blood flow, neutrophil infiltration, alteration of microvascular structures, and increase of acid and pepsinogen secretion. In addition, increased production of reactive oxygen species (ROS), increased lipid peroxidation, and neutrophil infiltration have demonstrated to play a role in the pathogenesis of NSAIDs-induced ulcers, including the aspirin-induced ulcer [6–11].

On the other hand, increased oxidative stress plays a key role in the pathogenesis of ethanol-induced gastric damage, so that as oral ethanol produces gastric mucosal lesions and erosions, it increases lipid peroxidation, raises hydroxyl radicals generation, and causes DNA damage, while it lowers the gastric content of reduced glutathione. Also, ethanol-induced ulceration is linked to reduced mucosa microcirculation and to increased apoptosis [12–16]. 

Vitamin C (VC) (ascorbic acid) is a water-soluble antioxidant that directly scavenges ROS, like superoxide and hydroxyl radicals, hydrogen peroxide, singlet oxygen, and hypochlorous acid, and also guarantees the chain-breaking antioxidant action of vitamin E (VE) by reducing the VE radical to VE [17, 18]. In addition, VC has been shown to attenuate aspirin-induced gastric damage and to offer effective gastroprotection [19, 20].

In turn, VE (alpha-tocopherol), a lipid-soluble antioxidant that scavenges ROS and functions as a chain-breaking antioxidant for peroxidation of membrane lipids, has been shown to inhibit neutrophils adhesion to endothelial cells and the oxygen production in activated neutrophils [21–23]. Antiulcer effects of VE on aspirin- and ethanol-induced ulcers have been reported [24–26].

Grape seed extracts (GSEs), rich in flavonoids, mainly proanthocyanidin, have been shown to produce effective antioxidant effects [22–30]. Experimental studies have shown that oral administration of GSE lowers ROS generation and plasma protein carbonyl groups, while it enhanced the activity of the endogenous antioxidant system [27–30]. Clinical trials have confirmed the antioxidant effects of GSE [31–33]. The antiulcer activity of GSE has been also referred [33].

In light of these issues and to our knowledge (Entrez PubMed review up to July 2011), no previous study compared the preventive effects of GSE, VC, and VE against aspirin- and ethanol-induced gastric ulceration and associated increases of lipid peroxidation; this study was aimed to do such comparison.

## 2. Materials and Methods

### 2.1. Animals

Male Sprague Dawley rats (200–250 g) from the National Center for Laboratory Animal Production (CENPALAB, Havana City, Cuba) were adapted to laboratory conditions (25 ± 2°C of temperature, 60 ± 5% of relative humidity, and 12-hour light/dark cycles) for 7 days. Food (rodent pellets from CENPALAB) and tap water were provided *ad libitum*. 

Experiments were conducted in accordance to the Cuban guidelines of Animal Handling and the Cuban Code of Good Laboratory Practices (GLP), which follow international guidelines for the use and care of laboratory animals. Study protocol and animal use were approved, prior to study beginning, by an independent Animal Ethics Committee.

### 2.2. Administration and Dosage

GSE (85% in proanthocyanidine) came from Blackmores (Sydney, Australia), and VC (Cuban Pharmaceutical Industry) was prepared as a suspension in acacia gum/water vehicle (1%), while VE (Carlson health, VIC, Australia), was suspended in a Tween 65/water suspension (2%). All suspensions were prepared daily, 1 hour before use and administered by oral gastric gavage (5 mL/kg of body weight) for 10 days. 

Two independent experiments were conducted, in which rats were randomized into eight groups: a negative control and seven groups that received aspirin or ethanol for ulcer induction: one orally treated with the vehicle (positive control) and the other six with 25 and 250 mg/kg of VC, VE, or GSE, respectively.

### 2.3. Experimental Induction of Gastric Ulcers

The animals were fasted for 24 hours before the experiments with free access to water.

In both experiments, rats were sacrificed in ether atmosphere and their stomachs were immediately removed for quantifying the lesions.

#### 2.3.1. Aspirin-Induced Gastric Ulcers

Following one hour of administering the last doses of vehicle, GSE, VC, or VE, a single oral dose of aspirin (300 mg/kg) was given by gastric gavage. Five hours later, rats were sacrificed.

#### 2.3.2. Ethanol-Induced Gastric Ulcers

We followed the procedure in accordance to Zengil et al. (1987) [34]. In brief, one hour after the last administering of the vehicle, GSE, VC, or VE, ethanol (60%) (1 mL/200 g body weight) was intragastrically administered to each rat. One hour later, rats were sacrificed.

### 2.4. Evaluation of Gastric Mucosal Damage

The stomachs were opened along the greater curvature and washed with saline solution. The lesions in the gastric mucosa were examined macroscopically using magnification 3x. Ulcer indexes were determined as the sum of the lengths of the whole gastric lesions (in mm). Two independent, blinded observers performed the observations and measurements of lesion lengths [35].

### 2.5. Determination of Lipid Peroxidation (LP) in Gastric Mucosa

Aliquots of gastric mucosa were obtained by gentle scraped with a scalp. Lipid peroxidation was assessed as per the content of thiobarbituric reactive substances (TBARS) in the gastric mucosa and quantified in accordance to Ohkawa et al. 1979 method [36], which has been widely used for this propose [37–40]. Results were expressed as nmol of malondialdehyde (MDA)/mg of protein. The protein concentration was determined according to modified Lowry method [41].

### 2.6. Statistical Analysis

Comparisons among groups were performed with the Kruskal Wallis test, and paired comparisons with the Mann-Whitney *U* test. The level of statistical significance was set at *α* = 0.05. All analyses were performed using Statistics software for Windows (Release 6.0, StatSoft; Inc, USA).

## 3. Results and Discussion

Oral administration of GSE, VC, and VE, all at 25 and 250 mg/kg, prevented aspirin- and ethanol-induced gastric mucosal ulceration and reduced the increase of gastric MDA elicited by these aggressive agents. To our knowledge (Entrez PubMed review up to July 2011), this study is the first comparative study of the gastroprotective effect of GSE with those of VC and VE against aspirin- and ethanol-induced ulceration and concomitant increase of lipid peroxidation on the rat gastric mucosa. 

No negative, but all positive controls exhibited typical aspirin-induced gastric ulcers. Aspirin increased significantly MDA gastric concentrations as compared to the negative controls (Table 1). Both doses of each treatment reduced the development of aspirin-induced gastric ulcers and attenuated the increase of gastric MDA.

Oral administration of VC, VE, and GSE 25 mg/kg decreased the ulcers (59.7%, 49.3%, and 57.3%, resp.) and gastric MDA (31.8, 52.8, and 63.6%, resp.) versus the positive controls, while, at 250 mg/kg, they produced ulcer reductions of 69.1%, 61.9%, and 56.3%, respectively, and MDA decreases of 67.4, 81.4, and 91.7%, respectively (comparisons versus positive control rats). The effect of the highest doses of each treatment revealed that the magnitude of ulcer reduction was comparable for all treatments, while MDA decrease with GSE was higher than with VC and tended to be greater, albeit non significantly (*P* = 0.058), than with VE. 

Oral administration of ethanol produced gastric ulceration and increased significantly the MDA gastric content (Table 2). All schemes protected against ethanol-induced gastric damage and reduced MDA gastric levels. Treatment with VC, VE, and GSE given at 25 mg/kg reduced ethanol ulcers (55.2%, 43.3%, and 81.6%, resp.) and attenuated MDA increases (45.2%, 43.4%, and 61.4%, resp.), while it at 250 mg/kg, reduced the ulcers by 54.8%, 59.6%, and 97.2%, respectively, and decreased MDA by 61.0%, 77.4%, and 83.2%, respectively, versus the positive controls. 

Oral treatment with GSE reduced ethanol-induced ulcers more effectively than VC and VE, while it produced reductions of MDA levels greater than VC, but similar to VE. The lowest dose of GSE, however, lowered MDA concentrations more than the same dose of VE. 

Overall, GSE 25 and 250 mg/kg produced greater percent reductions of ethanol than aspirin-induced ulcers. The reductions of gastric MDA levels with the lowest dose (25 mg/kg) of GSE were similar in both models, while the MDA reduction with the highest dose (250 mg/kg) seems to be greater in rats with aspirin-induced ulceration. 

The experimental models here used share similarities and differences. Increased oxidative stress and ROS production are pathogenic mechanisms of both models [4–9, 12]. Nevertheless, the key mechanism of NSAID-induced gastric ulceration results from the irreversible and nonselective inhibition of COX activity, which interferes with the synthesis from PG, triggering the effects derived of PG depletion, and shuttles the arachidonic acid metabolism towards the lipoxygenase pathway, increasing the formation of vasoconstrictor leukotrienes (LTs) [6, 7, 10, 11]. 

The gastric ulceration induced by oral administration of ethanol to rats involves other mechanisms in addition to the increase of oxidative stress and ROS formation. So, ethanol also depletes PG concentration due to its necrotizing action on the gastric mucosa and, thus, shares the consequences of PG as oral administration of aspirin does, as increased vascular permeability and decreased gastric mucosa microcirculation. Also, the necrotizing action of ethanol decrease, gastric mucus secretion and impairs the quality of mucus composition [42]. 

The protective effect of VC involves the reduction of lipid peroxidation in the gastric mucosa, which preserves the gastric microcirculation. VC stimulates the expression of the antioxidant and vasodilator heme oxygenase enzyme in the gastric epithelium and inhibits the expression of inducible nitric oxide synthase enzyme [9, 43, 44]. It makes sense, therefore, that VC had been effective to protecting against both aspirin- and ethanol-induced ulcers. The highest dose of VC reduced aspirin-induced ulcer more markedly (about 70%) than ethanol ulcers (about 55%), while the effects on gastric MDA (67% and 61%, resp.) were comparable. These results suggest that although the effects of VC against aspirin and ethanol ulcers may be attributable to the reduction of lipid peroxidation, this is not the only mechanism involved in the gastroprotective effect on aspirin-induced ulcers. Further studies must dilucidate the direct or indirect mechanism of action that supports the gastroprotective and antioxidant effects of Vitamin C.

Oral administered VE has been shown to be effective against ethanol and NSAIDs-induced ulcers, an action that involves the reduction of lipid peroxidation and the increases of the activity of endogenous antioxidant enzymes, like superoxide dismutase, catalase, and glutathione peroxidise [25–27]. Then, the fact that VE was effective in the two models here used was expected. The highest dose of VE produced similar reductions (about 60%) of aspirin- and ethanol-induced ulcers and of MDA values (roughly about 80%). Then, although these results suggest that the gastroprotective effects of VE may be related to its antioxidant effects, these last ones seem to be greater, a finding without conclusive explanation. 

The effect of VC for protecting against aspirin-induced ulcers (*≅*70% of inhibition) was apparently, not significantly, greater than that of VE (*≅*62% of inhibition), while the effects of both treatments on ethanol ulcers were grossly comparable. This result, however, is slightly different from that reported in the model of water-restrain-stress- (WRS-) induced ulcers, in which VE was more effective than VC, despite the fact that both substances exerted their protective actions through antioxidant and anti-inflammatory effects [45]. 

Oral administration of GSE protected against ethanol-induced gastric ulcers in rats more effectively than VE and VC, wherein the lowest dose of GSE produced a reduction of these ulcers (about 82%) and the highest dose practically abolished (about 97% reduction) the ethanol-induced ulcers. Although the decreases of MDA levels with GSE (*≅*83%) were greater than with VC (*≅*61%) or VE (*≅*77%), it should be noted that ethanol ulcer reduction with GSE was greater than MDA decreases, consistent with the fact that the protection of GSE against ethanol-induced ulcers depends not only on its scavenging properties, but on its ability for lowering increase MPO and consequent neutrophil infiltration [25, 46] as well. 

The magnitude of aspirin-induced ulcer reduction, comparable for all treatments, was apparently greater with VC 250 mg/kg (about 70%) than with the same dose of GSE (about 57%). By contrast, the same dose of GSE decreased MDA (about 97%) more markedly than VC (about 83.2%). These results suggest that although the efficacy of GSE on this model should be attributable to its ability to lower lipid peroxidation, it did not reduce other mechanisms leading to aspirin-induced ulcers.

## 4. Conclusions

GSE prevented ethanol-induced gastric ulcers more effectively than VC or VE, while its protection against aspirin ulcers was comparable for all treatments. GSE produced the greatest reductions of gastric MDA in both models. Then, the gastroprotective effects of GSE may be related, at least partially, to its ability for reducing lipid peroxidation in the gastric mucosa. Further studies must compare the therapeutic effects of GSE, VC, and VE on ethanol- and aspirin-induced gastric ulcer in rats.

## Figures and Tables

**Table 1:** Effects of GSE, VC, and VE on ulcer indexes and MDA gastric concentrations in rats with aspirin-induced ulcer.

Treatment	Doses (mg/kg)	Ulcer index (mm)	**I** (%)	MDA (nmol/mg of pt)	**I** (%)
Negative control (vehicle)	0	0 ± 0****	—	1.02 ± 0.21**	—
Positive control (vehicle + aspirin)	0	27.06 ± 5.60	—	12.28 ± 0.81	—
VC + aspirin	25	10.91 ± 2.33**	**59.7**	8.7 ± 0.28***	**31.8**
VC + aspirin	250	8.36 ± 1.23***	**69.1**	4.69 ± 0.92**	**67.4**
VE + aspirin	25	13.72 ± 3.60*	**49.3**	6.33 ± 0.86***	**52.8**
VE + aspirin	250	10.31 ± 2.70*	**61.9**	3.11 ± 0.43***	**81.4**
GSE + aspirin	25	11.56 ± 3.50*	**57.3**	5.11 ± 0.79^∗∗∗a^	**63.6**
GSE + aspirin	250	11.81 ± 3.03*	**56.3**	1.95 ± 0.28^∗∗∗at^	**91.7**

GSE: grape seed extract, VC: vitamin C, VE: vitamin E, MDA: malondialdehyde.

Data as means ± MSE (mean standard error).

*P < 0.05, **P < 0.01, ***P < 0.001; comparisons with positive controls, ^a^ *P* < 0.01; comparisons with the same dose of VC; ^t^ *P* = 0.054; comparisons with that of VE, (Mann-Whitney *U* test).

**Table 2:** Effects of GSE, VC, and VE on ulcer indexes and MDA gastric concentrations in rats with ethanol-induced ulcer.

Treatment	Doses (mg/kg)	Ulcer index (mm)	**I** (%)	MDA (nmol/mg of pt)	**I** (%)
Negative control (vehicle)	0	0 ± 0****	—	1.02 ± 0.21**	—
Positive control (vehicle + ethanol)	0	72.27 ± 8.43	—	18.72 ± 0.76	—
Vit C + ethanol	25	32.32 ± 10.1*	**55.2**	10.71 ± 0.54***	**45.2**
Vit C + ethanol	250	32.67 ± 8.8**	**54.8**	7.93 ± 0.88***	**61.0**
Vit E + ethanol	25	40.94 ± 9.6*	**43.3**	11.03 ± 0.57***	**43.4**
Vit E + ethanol	250	29.21 ± 9.8**	**59.6**	5.02 ± 0.59***	**77.4**
GSE + ethanol	25	13.27 ± 4.8^∗∗∗ab^	**81.6**	7.84 ± 0.45^∗∗∗aabb^	**61.4**
GSE + ethanol	250	2.03 ± 0.69^∗∗∗aab^	**97.2**	4.00 ± 0.28^∗∗∗aa^	**83.2**

GSE: grape seed extract, VC: vitamin C, VE: vitamin E, MDA: malondialdehyde.

Data as means ± MSE (mean standard error).

*P < 0.05, **P < 0.01, ***P < 0.001; comparisons with positive control,^a^ *P* < 0.05, ^aa^ *P* < 0.01 comparisons with the same doses of VC, ^b^ *P* < 0.05, ^bb^ *P* < 0.01 comparisons with the same doses of VE, (Mann-Whitney *U* test).
